# Clinical Application of Microvolume LC–MS/MS for Therapeutic Drug Monitoring of Immunosuppressants in Solid-Organ Transplant Recipients

**DOI:** 10.3390/jcm15041565

**Published:** 2026-02-16

**Authors:** Daiki Iwami, Natsuka Kimura, Sho Nishida, Makiko Mieno, Takehiro Ohyama, Kyoko Minamisono, Yasunaru Sakuma, Joji Kitayama, Yasushi Imai, Ryozo Nagai, Kenichi Aizawa

**Affiliations:** 1 Division of Renal Surgery and Transplantation, Department of Urology, Jichi Medical University, Shimotsuke 329-0498, Japan; 2 Department of Translational Research, Clinical Research Center, Jichi Medical University Hospital, Shimotsuke 329-0498, Japan; 3 Center for Information, Jichi Medical University, Shimotsuke 329-0498, Japan; 4 Department of General, Gastrointestinal, and Transplant Surgery, Jichi Medical University, Shimotsuke 329-0498, Japan; 5 Department of Pharmacy, Jichi Medical University Hospital, Shimotsuke 329-0498, Japan; 6 Jichi Medical University, Shimotsuke 329-0498, Japan; 7 Clinical Pharmacology Center, Jichi Medical University Hospital, Shimotsuke 329-0498, Japan

**Keywords:** therapeutic drug monitoring, microsampling, tacrolimus, mycophenolic acid, LC-MS/MS, hematocrit correction, solid-organ transplantation, pharmacokinetics

## Abstract

**Background/Objectives:** Therapeutic drug monitoring (TDM) is essential for optimizing immunosuppressive therapy in solid-organ transplant recipients by maintaining efficacy, while minimizing adverse effects. However, conventional TDM relies on venous sampling and separate assays for tacrolimus (TAC) in whole blood and mycophenolic acid (MPA) in plasma, thereby increasing patient burden and procedural complexity. To address these limitations, we investigated the clinical utility of a microvolume, liquid-phase microsampling device (MSW2™) in combination with liquid chromatography–tandem mass spectrometry (LC-MS/MS). **Methods:** We established and applied an LC-MS/MS method for simultaneous quantification of TAC, MPA, and mycophenolic acid β-D-glucuronide (MPAG) using only 2.8 µL of whole blood collected with MSW2™, which eliminates drying or extraction steps. Hematocrit-based correction was applied to estimate plasma MPA concentrations from whole-blood measurements. The method was evaluated in 60 renal transplant recipients with paired venous samples for comparison. Analytical performance was assessed using regression, Bland–Altman analyses, predictive metrics, and stability testing under different storage conditions. **Results:** Microsampled and venous concentrations were strongly correlated (R^2^ > 0.95). Estimated plasma MPA concentrations derived from whole blood closely approximated plasma concentrations (bias < 5%). Reducing the sample volume from 5.6 µL to 2.8 µL improved precision and increased the success rate of blood collection from 72.9% to 94.0%. All analytes remained stable for up to 72 h at ≤25 °C. **Conclusions:** This approach enables accurate, simultaneous quantification of multiple immunosuppressants from trace blood volumes. By reducing sampling burden and simplifying logistics, it provides a clinically feasible and patient-centered strategy for precision TDM, supporting broader implementation of limited sampling strategies and expanding applicability to pediatric, home-based, and telemedicine settings.

## 1. Introduction

Optimal dose adjustment of immunosuppressive drugs (IS), based on therapeutic drug monitoring (TDM), is essential to improve long-term prognosis after solid-organ transplantation (SOT) and to minimize risks of both hyper- and hypo-immunosuppression. The calcineurin inhibitor, tacrolimus (TAC), and an antimetabolite, mycophenolate mofetil (MMF), are employed in immunosuppressive protocols for more than 90% of kidney transplant recipients [1,2]. However, TAC and mycophenolic acid (MPA), the active immunosuppressive metabolite of MMF, exhibit a weak correlation between trough levels and pharmacological effects. Currently, the area under the concentration–time curve (AUC) derived from multiple blood samples is regarded as the optimal parameter for TDM. Accurate determination of the “true” area under the concentration–time curve (AUC) requires calculations based on multiple measured concentration–time points, typically eight or more, using the linear trapezoidal rule [3,4]. However, such intensive sampling is rarely practical in routine clinical settings. Therefore, limited sampling strategies (LSS) have been developed to estimate AUC using a reduced number of optimally selected sampling points, usually two to four, which show the strongest correlation with the full AUC. Furthermore, TAC and MPA concentrations are usually measured in blood and plasma, respectively, necessitating different collection tubes at each time point, thereby increasing the required blood volume and complicating the sampling procedure. As a result of these challenges, the AUC estimation method using LSS has not been widely utilized, despite its accuracy and efficacy in TDM [3,5,6].

Owing to technological innovations in mass spectrometry, high-performance liquid chromatography–tandem mass spectrometry (LC-MS/MS) now enables simultaneous quantification of multiple drug concentrations from blood volumes of ≤ 10 μL, which is less than 1% of the sample volume conventionally used in clinical laboratories [7]. The ability to measure multiple drug concentrations from such small volumes can address these challenges and promote a broader application of AUC estimation with LSS.

To reduce complexity and patient burden associated with repeated sampling, several microsampling tools, such as dried blood spots (DBS) [8,9,10] and volumetric absorptive microsamplers (VAMS) [11,12,13], have been developed and evaluated for their clinical utility. However, they entail multiple procedures, including drying, punching, and extraction, which discourage their widespread adoption in clinical practice.

Most recently, the microfluidic capillary microsampling device (MF-CMS), commercially available as MSW2™, has been developed to enable precise collection of very small blood volumes [14]. This device has an inlet for drawing blood into a U-shaped capillary microchannel coated with an anticoagulant, ethylenediaminetetraacetic acid disodium salt (EDTA-2Na). The liquid-phase blood sample simplifies measurements compared with using absorbents and dried samples.

In this study, we evaluated the performance of MSW2™ combined with LC-MS/MS for simultaneous quantification of multiple IS in a single blood microsample. Our goal was to establish a clinically applicable measurement platform that enables precise quantification of multiple immunosuppressants from liquid blood samples without drying steps, using minimal sample volumes and a streamlined workflow. This approach is expected to reduce the burden on both patients and clinical staff by replacing multiple conventional blood collections with simplified microsampling. The primary objective of this study was to evaluate the analytical and clinical performance of MSW2™ combined with LC–MS/MS for simultaneous measurement of tacrolimus, mycophenolic acid, and mycophenolic acid β-D-glucuronide using microvolume whole-blood samples. Secondary objectives were to assess the applicability of hematocrit-based correction for estimating plasma MPA concentrations, to compare analytical performance between two microsampling volumes (5.6 μL and 2.8 μL), to evaluate blood collection success rates, and to examine the stability of microsampled blood under various storage conditions. This approach is expected to reduce the burden on both patients and clinical staff by replacing multiple conventional blood collections with simplified microsampling, thereby facilitating more practical implementation of precision TDM.

## 2. Materials and Methods

### 2.1. Patients

Kidney transplant (KTx) patients being treated at Jichi Medical University Hospital for post-transplant follow-up care were invited to participate in this study. This study was conducted in accordance with the Declaration of Helsinki and approved by the Bioethics Committee for Clinical Research at Jichi Medical University Hospital (approval codes: CU20-064 and CU21-057; approval dates: 10 February 2021 and 1 February 2022, respectively). Written informed consent was obtained from all participants involved in the study. Therapeutic drug monitoring was performed approximately one month after kidney transplantation, corresponding to the clinical timing summarized in Table 1.

### 2.2. Capillary Microsampling Device

We employed an MF-CMS (Microsampling Fingerstick—Capillary Microsampling System, MSW2™, Shimadzu, Kyoto, Japan), purchased commercially, as the microsampling device. This device has an inlet port that drains blood (total volume 23 μL), filling a U-shaped capillary coated with an anticoagulant, EDTA-2Na (Supplementary Figure S1). The patient’s fingertip was pricked with a 30-G lancet and blood was collected. After the capillary was filled with blood, an H-shaped segment was “snapped” from the main channel, and used for analysis. MSW2™ allows precise collection of predefined blood volumes (either 5.6 µL or 2.8 µL) by adjusting the folding position of the device. The 5.6-µL and 2.8-µL microsamples were collected in separate sampling events, and no paired samples of different volumes were collected from a single puncture. The amount of blood collected can also be determined by filling either one side of the U-shaped blood collection channel or by filling the entire channel. Collection of 5.6 μL of blood can be seen in Movie S1 and of 2.8 μL in Movie S2. The process of snapping off the microsampling segment and storing it in an Eppendorf tube can be seen in Movie S3. All microsampling procedures were performed by professional phlebotomists in the hospital. Plomley et al. provided proof of concept that the MF-CMS was able to achieve precision of 8.0% and accuracy from 90.9 to 110% across a range of samples for centanafadine and its lactam metabolite [15].

### 2.3. Simultaneous Measurement of TAC and MPA Concentrations in Blood Using the Same Microsample and a Correction for Hematocrit

TAC and MPA measurements were performed using validated LC-MS/MS. Assay methodology, limits of quantification for each analyte, and quality control results have been published previously [1,2]. The limits of detection (LOD) of the LC–MS/MS method were 0.1 µg/mL for mycophenolic acid (MPA), 1 µg/mL for mycophenolic acid β-D-glucuronide (MPAG), and 1.73 ng/mL for tacrolimus (TAC). The same validated analytical method was applied to both capillary microsamples (2.8 µL and 5.6 µL) and venous blood samples; therefore, LOD values were identical regardless of sampling volume or collection method. Immunosuppressants in whole-blood samples were quantified according to the following procedure: Whole blood from patients collected using MSW2™ was immediately divided (Figure S1) and stored in microtubes at −80 °C until analysis. On the day of analysis, 5.6 µL or 2.8 µL of whole blood were obtained by centrifugation from the thawed whole blood in the tube. This whole blood was mixed with internal standards for TAC and MPA and 200 µL of extraction buffer, vortexed for 60 s, and then centrifuged. Approximately 100 µL of the supernatant extract was transferred to an LC-MS/MS analysis vial, and 20 µL of the extract was injected into the analytical system. Simultaneous analysis of immunosuppressants was performed using an LCMS-8050 triple quadrupole mass spectrometer (Shimadzu, Kyoto, Japan).

### 2.4. Measurement Concordance of Blood Samples from Venipuncture and from Fingertip Capillary Microsampling

At the time of microsampling with MSW2™ from fingertips of patients, an additional 2 mL of blood was collected by venipuncture and separated into whole-blood and plasma samples. Tacrolimus (TAC) and mycophenolic acid (MPA) concentrations were measured in these specimens by LC-MS/MS using our previously developed and validated method [1,2].

To evaluate whether MPA concentrations measured in whole blood could be reliably converted to estimated plasma values, an established hematocrit (Hct)-based correction formula was applied to whole-blood MPA concentrations to estimate plasma MPA concentrations (estimated plasma MPA = blood MPA × 100/(100 − Hct (%))) [16]. Hct values were obtained from venous blood samples measured on the same day.

For clarity, the term “estimated plasma MPA” is used throughout this manuscript to denote plasma MPA concentrations estimated from whole-blood measurements using Hct correction, rather than directly measured plasma MPA.

Although the amount of capillary blood collected is very small, the success rate of collecting the required amount of blood is an important concern in clinical practice. On the other hand, if the volume of collected blood is too small, inaccuracy of the concentration measurement is not negligible. In other words, forcibly squeezing the fingertips to collect the required amount of blood increases the risk of contamination with exudates. Thus, blood was collected simultaneously with regular venipuncture and microsampling with MSW2™ from patient fingertips. Concentrations in the two volumes of blood (5.6 µL and 2.8 µL) collected with MSW2™ were compared with those from venipuncture blood.

### 2.5. Microsample Stability in MSW2™ Under Various Storage Conditions

Blood samples used for stability experiments were obtained from 20 kidney transplant recipients. We examined stability of blood microsamples kept under various storage conditions, assuming that patients would bring their self-collected blood samples to the hospital or send them by mail. An additional 2 mL of blood were drained with MSW2™ resulting in 12 aliquots of snapped MSW2™ (5.6 μL) that were stored in Eppendorf tubes at various temperatures (−30 °C, 4 °C, 25 °C, and 60 °C) and for various storage periods (24 h, 48 h, 72 h) until measurement. Then, concentrations of TAC, MPA, and MPAG were simultaneously measured in each sample and results were compared to determine conditions under which microsampled blood remains stable in an ordinary transportation environment. Drug concentration ratios at each temperature and for each preserved period were compared with that of 0 h blood using Friedmann analysis. The Friedman test is a non-parametric statistical test used to detect differences in three or more related (paired) groups [17]. It is the non-parametric alternative to repeated measures ANOVA.

### 2.6. Statistical Analysis

Comparison of immunosuppressant concentrations measured in blood samples collected by venipuncture and those collected using the MSW2™ device was performed using Passing–Bablok regression and Kendall’s tau (τ), a non-parametric measure of rank correlation that assesses the strength and direction of the association between two variables. For pharmacokinetic evaluation in therapeutic drug monitoring, the area under the concentration–time curve from 0 to 12 h (AUC_0–12_) was used for mycophenolic acid (MPA), whereas the area under the concentration–time curve from 0 to 24 h (AUC_0–24_) was used for tacrolimus (TAC), in accordance with established clinical practice. Bias was analyzed using the Bland–Altman method. In addition, the success rate of collecting required blood volumes (5.6 μL and 2.8 μL) was calculated and compared by chi-square test. The predictive performance of microsampled blood compared with venipuncture was tested using a method previously reported [18]. IS drug concentrations were also compared for bias and imprecision. Bias was assessed by calculating the mean prediction error (MPE), and imprecision was estimated by calculating the root mean squared error (RMSE) and the mean absolute percentage error (MAPE). Acceptable values for MAPE were set at <15% [19]. To assess stability of microsampled blood stored in snapped segments of MSW2™ in various environments, concentrations of TAC, MPA, and MPAG were evaluated with Friedman analysis. We used JMP Pro 17 (SAS Institute Inc., Cary, NC, USA) or Graphpad PRISM 10 (GraphPad Software, LLC.) for statistical analysis. *p*-values less than 0.05 were considered significant for differences between groups. Normality of TAC and MPA concentrations was assessed using the Shapiro–Wilk test. Because distributions did not meet the assumption of normality, these variables are presented as medians with interquartile ranges (25th–75th percentile) instead of means ± standard deviations.

## 3. Results

### 3.1. Patients Demographics

In total, 60 kidney transplant recipients participated in the current study. Patient characteristics are shown in Table 1. Mean age at transplant was 46.2 ± 12.6 years, and 48.3% (29 cases) of participants were male. The pre-transplant dialysis period was 39.9 ± 75.8 months (pre-emptive kidney transplant 16 cases, 26.6%). There were 18 diabetic recipients (30.0%) in the cohort. Mean post-transplant period at enrollment into the study was 4.2 ± 4.0 years. All patients were maintained with TAC and MMF with or without methylprednisolone (mPSL) (52 cases with mPSL and 8 cases without). There were 11 cases (18.3%) under everolimus (EVR). Mean daily dosages of TAC and MMF were 2.9 ± 1.7 mg/day and 916.7 ± 384.2 mg/day, respectively. Mean Hct was 34.95 ± 6.05%. Mean kidney allograft function was 1.5 ± 1.2 mg/dL of serum creatinine and 44.6 ± 14.6 mL/min/1.73 m^2^ of eGFR.

### 3.2. Mycophenolic Acid Concentrations in Blood Are Correctable to Those in Plasma Using the Hematocrit Value

**Figure 1:**
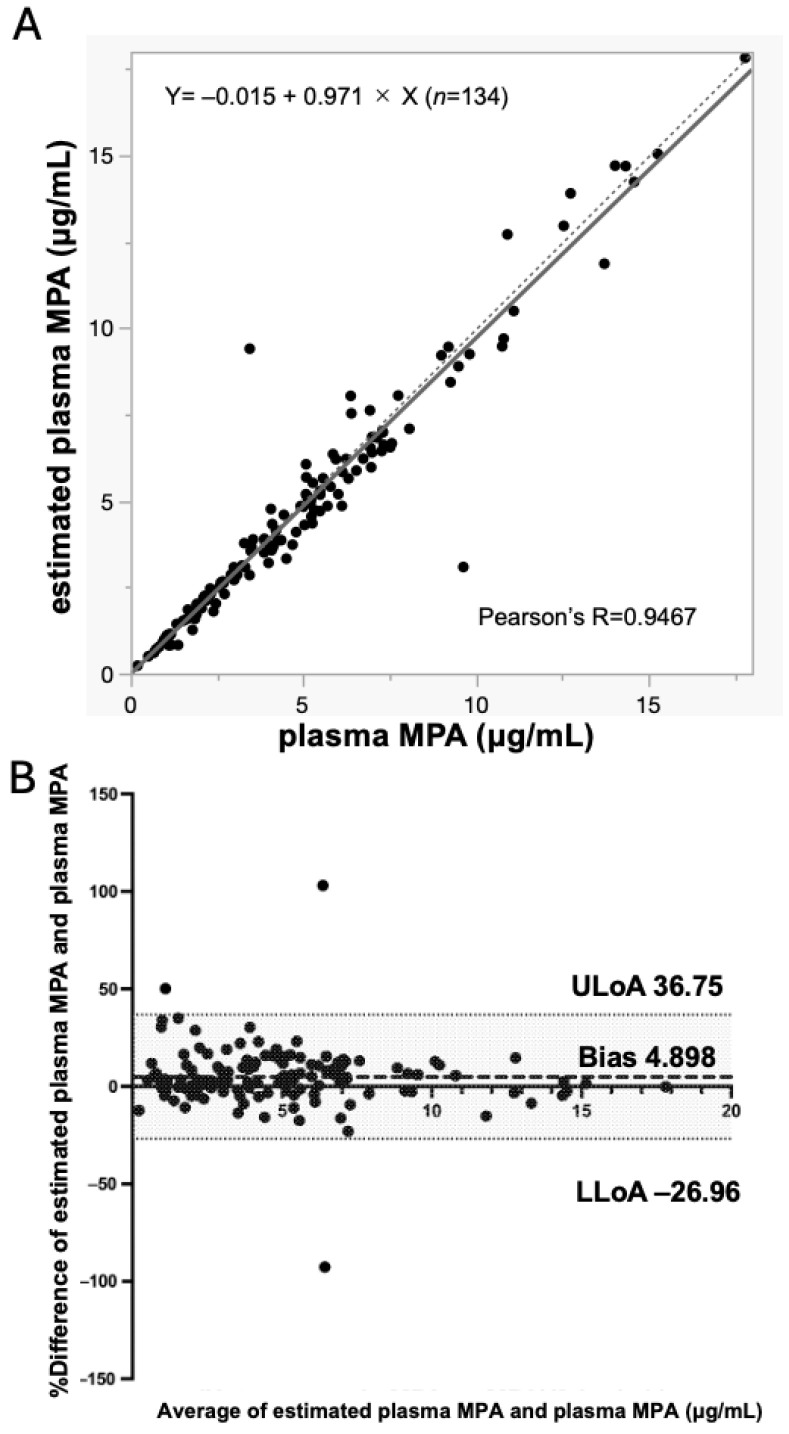
Conversion of whole-blood MPA to estimated plasma MPA using hematocrit correction. Venous blood collected from kidney transplant recipients was separated into whole blood and plasma, and mycophenolic acid (MPA) concentrations were measured by LC-MS/MS in both specimens. Whole-blood MPA concentrations were converted to estimated plasma MPA concentrations using the following hematocrit-based correction formula: [Estimated plasma MPA = whole-blood MPA × 100/(100 − Hct)]. Passing–Bablok regression analysis was performed to assess agreement between estimated plasma MPA and directly measured plasma MPA concentrations ((**A**), *n* = 134). The solid line represents the regression line. The dotted line represents the line Y=X. Bland–Altman analysis was used to evaluate bias between estimated plasma MPA and measured plasma MPA concentrations ((**B**), *n* = 134). Thick and thin dashed lines represent bias and limits of agreement, respectively. Abbreviations: Hct, hematocrit; LLoA, lower limit of agreement; MPA, mycophenolic acid; ULoA, upper limit of agreement.

First, the correlation between blood MPA and plasma concentrations was determined. The agreement between whole-blood MPA and plasma MPA after Hct correction has been demonstrated previously (Figure 1 [2]). In the present study, whole-blood MPA concentrations were converted to estimated plasma MPA concentrations using hematocrit values, and the relationship between estimated plasma MPA and directly measured plasma MPA concentrations was evaluated.

Passing–Bablok regression analysis between estimated plasma MPA and plasma MPA showed a systematic difference of 1.5% and a correlation coefficient of 0.971 (0.940–1.002), with a Kendall’s τ value of 0.884 (Figure 1A and Supplementary Table S1).

Bland–Altman analysis showed an average difference of 4.898% with 95% limits of agreement ranging from −26.96% to 36.74% (Figure 1B and Supplementary Table S1). These results demonstrate that whole-blood MPA concentrations can be reliably converted to estimated plasma MPA concentrations using hematocrit correction, enabling accurate comparisons with TAC measured in whole blood. On the basis of this validated conversion, we subsequently performed integrated analyses of tacrolimus, MPA, and MPAG concentrations using whole-blood samples to allow direct comparisons between venipuncture and capillary microsampling.

### 3.3. Blood Drawn by Venipuncture and That Microsampled with MSW2^TM^ Demonstrated the Same Values for TAC, MPA, and MPAG

**Figure 2:**
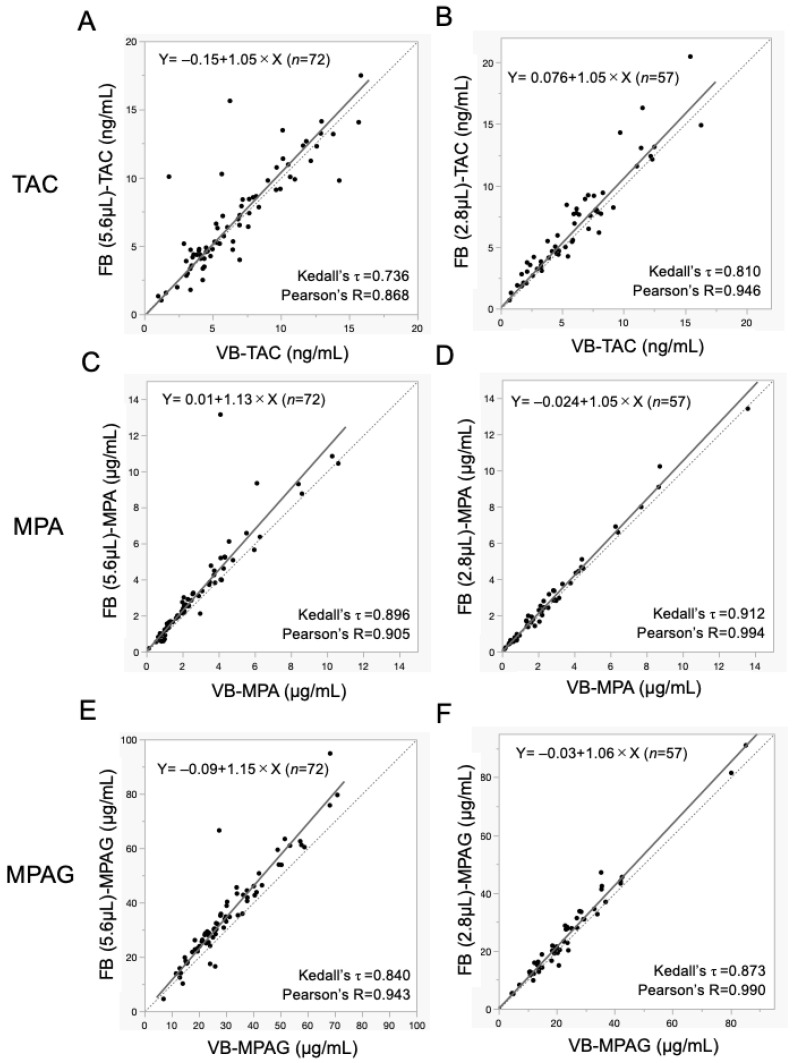
Passing–Bablok regression between blood microsampled with MSW2™ and venous blood for immunosuppressive drug concentrations. Concentrations of tacrolimus (TAC), mycophenolic acid (MPA), and mycophenolic acid β-D-glucuronide (MPAG) were measured in microsampled blood (5.6 μL or 2.8 μL) collected from fingertips using MSW2™ and in venous blood. Passing–Bablok regression analysis was performed for 5.6-μL microsamples ((**A**,**C**,**E**); *n* = 72) and for 2.8-μL microsamples ((**B**,**D**,**F**); *n* = 57) compared with venous blood. Solid lines represent the regression lines for each regression equation. Dotted lines represent the line of identity (Y = X). Abbreviations: FB: finger-pricked blood, MPA: mycophenolic acid, MPAG: mycophenolic acid β-D-glucuronide, TAC: tacrolimus, VB: venipuncture blood.

**Figure 3:**
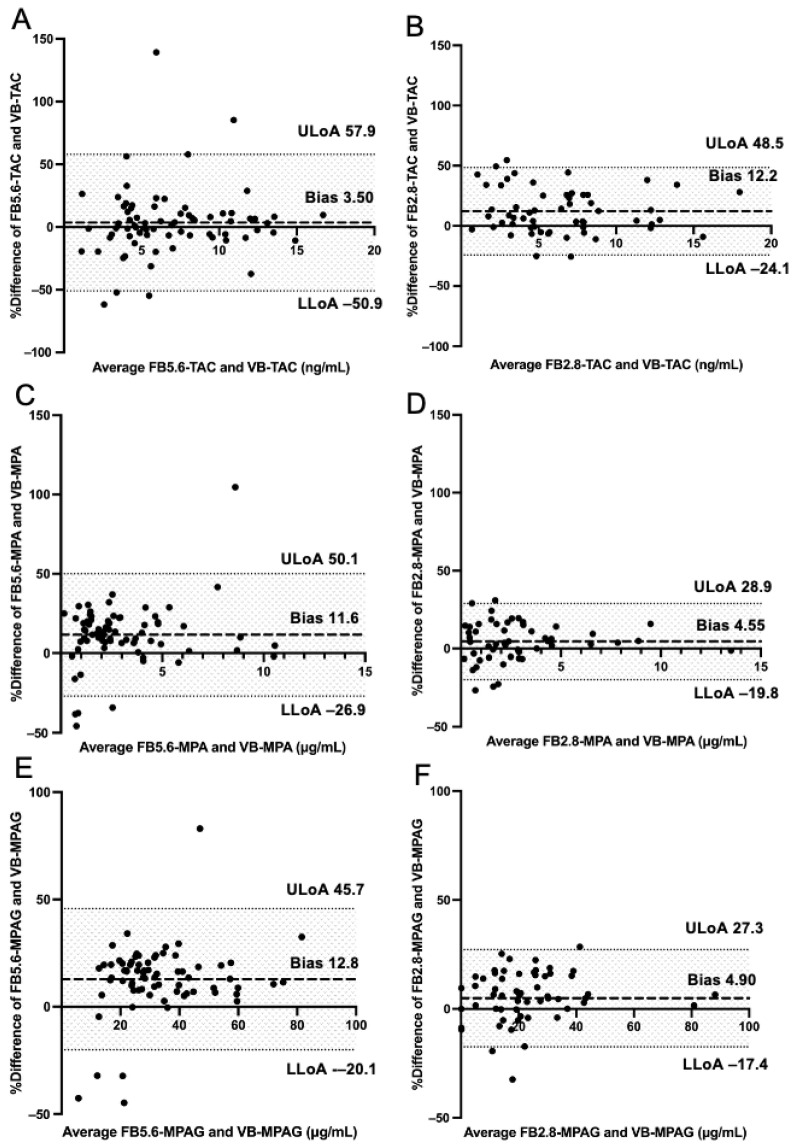
Bland–Altman analysis of venous blood samples and microsamples collected with MSW2™ for immunosuppressive drugs. Concentrations of TAC, MPA, and MPAG were measured in microsamples collected from fingertips with MSW2™ (5.6 µL or 2.8 µL) and in venous blood samples. Bland–Altman analysis was performed to analyze bias. Panels (**A**,**C**,**E**) correspond to 5.6-µL microsamples (*n* = 72) for TAC, MPA, and MPAG, respectively, compared with venipuncture-derived whole blood. Panels (**B**,**D**,**F**) correspond to 2.8-µL microsamples (*n* = 57) for TAC, MPA, and MPAG, respectively. Thick and thin dashed lines represent bias and limits of agreement, respectively. Abbreviations: FB: finger-pricked blood, MPA: mycophenolic acid, MPAG: mycophenolic acid β-D-glucuronide, TAC: tacrolimus, VB: venipuncture blood.

Results of Passing–Bablok regression analysis examining the correlation between analyte concentrations measured through MSW2^TM^ and venipuncture are shown in Table 2 and Figure 2. By reducing the volume of blood collected with MSW2^TM^ from 5.6 μL to 2.8 μL, Passing–Bablok regression analysis showed a slope approaching 1 and an intercept approaching 0 for all TAC (Figure 2A,B), MPA (Figure 2C,D) and MPAG values (Figure 2E,F). By reducing the sample volume, τ values improved from 0.736 to 0.810 for TAC, from 0.896 to 0.912 for MPA, and from 0.840 to 0.873 for MPAG. Results of the Bland–Altman analysis are shown in Table 2, with percentage difference-versus-average plots displayed in Figure 3. Reduction in the volume of blood collected resulted in decreased bias in Bland–Altman analysis, and demonstrated a narrower confidence band of limits of agreement for TAC (Figure 3A,B), MPA (Figure 3C,D), and MPAG (Figure 3E,F). These results indicate that finger-pricked 2.8 μL samples have higher accuracies than 5.6 μL samples.

Results for the predictive performance of MSW2^TM^ collecting 5.6 μL and 2.8 μL compared with venipuncture are summarized in Table 2. Although a total of 60 kidney transplant recipients were enrolled in this study, the number of analyzable microsamples differed between sampling volumes owing to differences in blood collection success. Specifically, 70 5.6-µL microsamples were initially attempted, of which 51 were successfully collected and included in the analysis, whereas 50 2.8-µL microsamples were attempted, of which 47 were successfully collected. These successful microsamples were paired with corresponding venous blood samples obtained at the same time for comparative analysis.

The MPE associated with collection of 5.6-μL blood samples versus those collected by venipuncture was 1.101 ng/mL for TAC, 0.537 μg/mL for MPA, and 5.452 μg/mL for MPAG. The MPE associated with 2.8-μL samples compared with venipuncture was 0.932 ng/mL for TAC, 0.239 μg/mL for MPA, and 2.322 μg/mL for MPAG measurements. Values for RMSE were all lower in TAC, MPA, and MPAG for 2.8 μL samples compared with 5.6-μL samples, representing a good correlation (high precision) between the two sample volumes. MAPE associated with collecting 5.6 μL versus venipuncture was 16.599%, 15.959%, and 16.128% for TAC, MPA, and MPAG, respectively. MAPE associated with collecting 2.8-μL samples compared with those collected by venipuncture was 14.920%, 12.267%, and 10.129% for TAC, MPA, and MPAG, respectively. These data demonstrated that by reducing the collected volume from 5.6 to 2.8 μL, all MAPE values improved from above 15% to below 15%.

Consistent with these findings, blood collection success improved significantly when the sampling volume was reduced, from 72.9% (51 successes and 19 failures) for 5.6-µL samples to 94.0% (47 successes and 3 failures) for 2.8-µL samples (*p* = 0.0032; Supplementary Table S2), indicating that reducing the collected blood volume not only enhanced analytical accuracy, but also substantially increased the feasibility of microsampling in clinical practice.

### 3.4. Stability of Microsampled Blood at Various Temperatures

**Figure 4:**
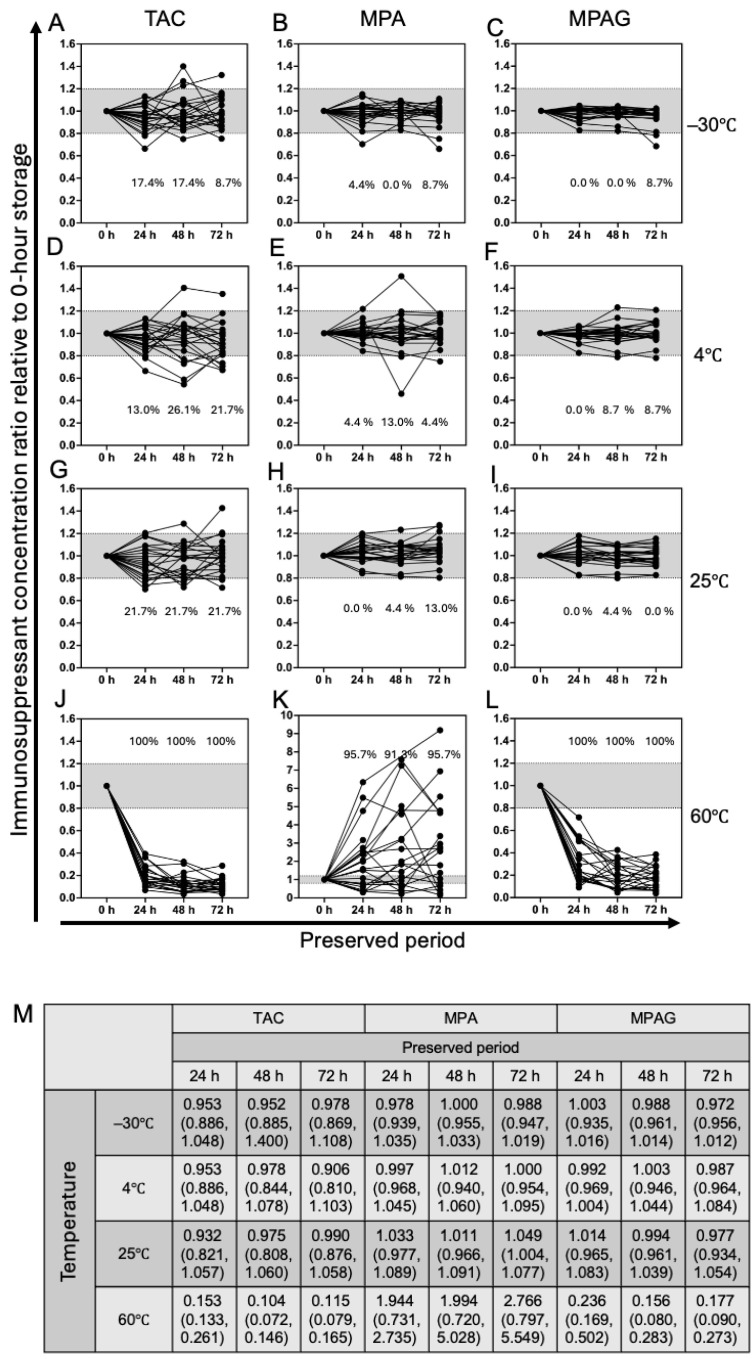
Stability of TAC, MPA, and MPAG concentrations in microsampled whole blood under various temperature and storage conditions. (**A**–**L**) Stability of microsampled anticoagulated whole blood in snapped MSW2™ segments was examined after storage at various temperatures (−30 °C, 4 °C, 25 °C, and 60 °C) and for various storage periods (24 h, 48 h, and 72 h) (*n* = 20). The *y*-axis indicates the immunosuppressant concentration ratio relative to 0 h storage, and the *x*-axis indicates storage duration. Changes in TAC, MPA, and MPAG concentrations relative to 0 h were evaluated up to 72 h under the four storage temperatures using the Friedman test. Numbers shown in each panel represent the frequency with which the measured concentration deviated by more than ±20% from the corresponding 0 h value. The gray area indicates a range of ±20% relative to the original measurement value. In panel K, seven data points exceeded the upper limit of the *y*-axis and are therefore not displayed within the plotting area. (**M**) Quantitative summary for each analyte. Values are shown as median (25th–75th percentile). Abbreviations: TAC, tacrolimus; MPA, mycophenolic acid; MPAG, mycophenolic acid β-D-glucuronide.

Microsampled blood collected from patients was stored at various temperatures (−30 °C, 4 °C, 25 °C, and 60 °C) and with various storage periods (24 h, 48 h, and 72 h) to determine sample stability. Concentrations of TAC, MPA, and MPAG were determined and the ratio to 0 h blood samples did not change significantly at −30 °C, 4 °C, or 25 °C for up to 72 h (Figure 4A–I,M). In contrast, TAC, MPA, and MPAG concentrations at 60 °C changed markedly during 72 h, with a % median reduction (concentration ratio) of 88.5% (0.115) for TAC and 82.3% for MPAG (0.177), and a 176.6% increase (2.766) for MPA (Figure 4J–M). These results indicate that microsampled blood should be stored and transported at <25 °C (Figure 4 and Supplementary Figures S2–S4).

## 4. Discussion

In this study, we utilized MSW2™, a blood microsampling device with the unique capability of storing and measuring liquid samples. Our findings demonstrate not only precision in concentrations comparable to those of other microsampling devices, but also remarkable success in blood collection by expert nurses. Furthermore, microsampled blood remained stable for up to 72 h. These results underscore the substantial potential of MSW2™ as a practical clinical tool, particularly in the context of telemedicine and precision pharmacotherapy.

At the beginning of the study, we sought to establish a system in which concentrations of TAC and MPA could be measured with the same blood sample. Historically, MPA concentrations have been measured in plasma, whereas TAC has been measured in anticoagulated whole blood, due to their different distribution properties in blood [20]. Simultaneous TAC and MPA measurements have been reported [3,5]; however, it is necessary to separate anticoagulated whole-blood samples into whole blood and plasma for separate measurements, which is tedious and time-consuming. In the present study, we demonstrated that MPA concentrations measured in whole blood can be reliably converted to estimated plasma MPA concentrations using hematocrit (Hct) correction. As shown in Figure 1, whole-blood MPA concentrations corrected for Hct showed high concordance with directly measured plasma MPA concentrations, as evidenced by Passing–Bablok regression and Bland–Altman analyses. Thus, this correction approach is robust and clinically applicable. These findings are consistent with previous reports [6,19], supporting the validity of estimating plasma MPA concentrations from whole-blood measurements. Our results further indicate that routine plasma separation for MPA monitoring may be omitted in clinical practice, thereby simplifying sampling procedures and reducing patient burden.

Stability of TAC, MPA, and MPAG in blood stored in snapped MSW2^TM^ segments was confirmed for up to 72 h, at −30 °C, 4 °C, and 25 °C without degradation but not at 60 °C. Interestingly, our analysis revealed a significant reduction in MPAG (down to one-tenth) at 60 °C, accompanied by a marked increase in MPA, suggesting potential disruption of glucuronic acid conjugation at elevated temperatures, leading to conversion of MPAG into unconjugated MPA. As discussed by Scuderi and colleagues [19], MPAG is an inactive substance in terms of immunosuppression, so its measurement is of little clinical significance. However, the present study revealed the possibility that glucuronic acid conjugates may degrade, releasing unconjugated MPA, depending on storage conditions. Certain analytes exhibit enhanced stability in dried formats due to limited enzymatic or hydrolytic degradation. Nevertheless, under clinically relevant conditions, we confirmed that all analytes remained sufficiently stable, supporting the practical feasibility of MSW2™ for routine TDM in real-world settings. Initially, we were concerned that liquid samples would prove unstable; however, our study showed that if stored at 25 °C or below, samples are stable for at least 72 h. If longer storage is required, it may be useful to place folded MSW2™ segments in an Eppendorf tube in a storage solvent at the time of blood collection. This flexibility of handling and transport further underscores the potential of MSW2™ for decentralized and patient-centered monitoring strategies.

While most samples demonstrated strong correlations, a few outlying values were observed. These outliers may be attributed to technical issues during sample collection or handling, such as insufficient blood volume or contamination with exudates. Further investigation into these occurrences is warranted to ensure reliability of the microsampling procedure. Furthermore, the observed improvement in accuracy with 2.8 μL samples compared with 5.6 μL samples may be attributed to multiple factors.

Although smaller sample volumes are generally considered more susceptible to dilution by interstitial fluid, our results showed a tendency toward higher measured concentrations in the 5.6 µL samples than in the 2.8 µL samples. This suggests that the observed variability was not primarily driven by dilution effects in the 2.8 µL samples, but rather by pre-analytical factors affecting larger-volume collections.

Collecting 5.6 µL requires a longer sampling time and a larger total blood volume to fill the entire U-shaped capillary, which may allow partial drying of the fingertip blood droplet prior to complete capillary filling. Such evaporation can lead to localized hemoconcentration and occasional overestimation of analyte concentrations. In addition, prolonged manual pressure during collection may further promote hemoconcentration rather than dilution.

Collecting smaller blood volumes reduces the risk of contamination from exudates and hemolysis. Additionally, smaller volumes minimize the need for manual pressure during collection, which in turn lowers the risk of introducing air bubbles into samples. These considerations suggest that the 2.8-μL sampling format represents an optimal balance between patient comfort, sampling success rate, and analytical accuracy.

In the present study, precision was defined as the reproducibility of analyte concentrations obtained from paired microsampled capillary blood and venous blood collected under standardized clinical conditions. Precision was evaluated using regression analyses, Bland–Altman analysis, and predictive performance metrics, including the mean prediction error, root mean squared error, and mean absolute percentage error. Repeated or simultaneous capillary samplings from different fingers of the same patient were not performed, and potential variability related to dilution with interstitial fluid was therefore not directly assessed. All microsampling procedures were conducted by trained healthcare professionals, and sampling by patients themselves was not evaluated in this study. These aspects represent limitations of the present work and should be addressed in future studies.

Recent technological advances have led to the development of micro-blood sampling devices such as DBS [11] and VAMS [10,21,22], which allow quantitative and qualitative evaluation of target substances with relative ease. However, using these devices poses challenges in accurately quantifying analytes, involving complex operations. DBS requires careful handling for accurate measurements and optimization adjustments, as per earlier studies and FDA guidelines [23]. DBS and VAMS require intricate extraction steps to convert dried phases into liquid samples for measurement. By contrast, MSW2™ enables direct liquid-phase measurement with minimal handling and simpler, more precise quantification of blood volume achieved by adjusting the folding position of the device. These combined features make MSW2™ particularly suitable for widespread clinical implementation. Although there were concerns about sample instability with storage in a liquid state, samples proved stable for at least 72 h at room temperature or lower with the stipulation that snapped segments are stored in a tube. Handling samples in a liquid state is particularly advantageous for clinical testing. Compatible with both blood and plasma, MSW2™ proved versatile for TDM and biomarker measurements beyond immunosuppressive drugs.

Capillary sampling with MSW2™ can efficiently quantify microfluidic samples for antibiotic concentrations in small blood volumes [15], comparable to amounts needed for self-measuring blood glucose (less than 5 μL). For instance, VAMS requires approximately 10–30 μL of blood, whereas MSW2™ requires only 5 μL, a significant difference. Notably, there is a marked difference in success rate between 5.6 μL and 2.8 μL. Specifically, collecting 5.6 μL requires a total volume of 23 μL to fill the entire channel of MSW2™, whereas collecting 2.8 μL requires as little as 5 μL to fill one side of the channel, due to the U-shaped structure of the channel. Saito et al. reported precise measurement of a drug of interest (Lenvatinib) in blood collected with MSW2™ [24]. However, they used 5.6-µL samples, and our study is the first report to demonstrate the usefulness of 2.8-µL samples in a clinical setting. This novel finding highlights the clinical applicability of MSW2™ for small-volume patient populations, including pediatric and transplant recipients, for whom minimizing blood loss is crucial. These results will strongly encourage telemedicine utilizing microsampling from fingertips, heels, and earlobes by patients themselves. By using only one side of a U-shaped capillary, blood collection success will increase. Moreover, accuracy of drug measurements will also be improved.

Future research should include collection of microsamples of blood by patients themselves so as to evaluate the success rate of self-collection, as well as further evaluation to determine whether precise concentration measurement is possible after sending blood samples by airmail. An improved version of MSW2™, achieved by modifying the shape of the blood collection tube, will reduce the part of the device in which folding the device wastes blood. Such an improvement enables microsampling down to a few µL, a crucial enhancement for practical applications.

Although the MSW2™ device used in this study is currently designated for research use only and has not yet been approved as a medical device under the Pharmaceuticals and Medical Devices Act in Japan or equivalent regulatory organizations elsewhere, the manufacturer is actively pursuing regulatory registration. Our study was conducted under IRB approval and in accordance with ethical guidelines to demonstrate the feasibility and accuracy of microsampling for therapeutic drug monitoring in a clinical research context. By validating its application in real-world clinical practice, we seek to support its progress toward regulatory approval and broad clinical adoption. Clinical advantages of MSW2™, including minimal invasiveness, reduced sample volume, and compatibility with remote care, make it a promising candidate for the future of patient-centered TDM.

In conclusion, we have demonstrated the feasibility and reproducibility of determining IS concentrations from 2.8-µL blood samples, which can be obtained from fingertip skin-pricks. This practical innovation has the potential to broaden access to AUC-based TDM and to improve clinical outcomes in SOT recipients through more precise and patient-friendly drug exposure assessments.

## Figures and Tables

**Table 1:** Patient characteristics.

Total Cases 60 Recipients		
Sex	Male: Female = 29 (48.3%): 31 (51.7%)	
Donor type	Living: Deceased = 59 (98.3%): 1 (1.67%)	
Age at transplant (years)	46.2 ± 12.6	
Time after transplant (years)	4.2 ±4.0	
Height (cm)	163.5 ± 8.3	
Body weight (kg)	62.8 ± 14.1	
BMI	23.7 ± 4.4	
Donor age at KTx	58.6 ± 11.5	
Diabetes Mellitus	18 cases (30.0%)	
Pre-transplant dialysis period (months)	39.9 ± 75.8 (pre-emptive 16 cases)	
Pre-transplant Dialysis modality	None: PD: HD = 16: 8: 36	
Immunosuppression	TAC, MMF, mPSL	46 cases (76.6%)
	TAC, MMF, mPSL, EVR	6 cases (10.0%)
	TAC, MMF, EVR	5 cases (8.3%)
	TAC, MMF	3 cases (5.0%)
TAC dose (mg/day)	2.9 ± 1.7	
MMF dose (mg/day)	916.7 ± 384.2	
TAC concentration (ng/mL), median (25th–75th percentile)	6.90 (4.60–10.20)	
MPA concentration (μg/mL), median (25th–75th percentile)	5.30 (3.15–7.40)	
Hct value at sampling (mg/dL)	34.95 ± 6.05	
sCr (mg/dL)	1.5 ± 1.2	
eGFR (ml/min/1.73 m^2^)	44.6 ± 14.6	

Abbreviations: BMI: body mass index, EVR: everolimus, Hct: hematocrit, HD: hemodialysis, KTx: kidney transplantation, MMF: mycophenolate mofetil, MPA: mycophenolic acid, mPSL: methylprednisolone, PD: peritoneal dialysis, sCr: serum creatinine, TAC: tacrolimus.

**Table 2:** Comparison of blood microsampled using MSW2™ and that which was drawn by venipuncture.

	Comparison	Passing–Bablok Regression			Bland–Altman Ratio % Difference vs. Average		Predictive Performance		
		Slope (95% CI)	Intercept (95% CI)	τ Value	Bias (95% LOA)	Bias SD	MPE	RMSE	MAPE (%)
TAC	FB (5.6 μL) vs VB	1.052 (0.964 to 1.131)	−0.157 (−0.609 to 0.281)	0.736	3.50 (−24.1 to 48.5)	27.8	1.101	0.654	16.599
	FB (2.8 μL) vs VB	1.048 (0.964 to 1.168)	0.075 (−0.304 to 0.551)	0.810	12.2 (−50.9 to 57.9)	18.5	0.932	0.488	14.920
MPA	FB (5.6 μL) vs VB	1.128 (1.064 to 1.209)	0.006 (−0.125 to 0.1099)	0.896	11.6 (−26.9 to 50.1)	19.7	0.537	0.270	15.959
	FB (2.8 μL) vs VB	1.053 (1.016 to 1.105)	−0.016 (−0.130 to 0.032)	0.912	4.55 (−19.8 to 28.9)	12.4	0.239	0.181	12.267
MPAG	FB (5.6 μL) vs VB	1.150 (1.081 to 1.233)	−0.091 (−2.041 to 2.004)	0.840	12.8 (−20.1 to 45.7)	16.8	5.452	4.227	16.128
	FB (2.8 μL) vs VB	1.065 (1.035 to 1.103)	−0.027 (−0.598 to 0.362)	0.873	4.90 (−17.4 to 27.3)	11.4	2.322	1.618	10.129

Abbreviations: FB: finger-pricked blood, CI: confidence interval, LOA: limit of agreement, MAPE: mean absolute percentage error, MPA: mycophenolic acid, MPAG: mycophenolic acid β-D-glucuronide, MPE: mean prediction error, RMSE: root mean squared error, TAC: tacrolimus, VB: venipuncture blood.

## Data Availability

All data are available in the main text or the Supplementary Materials.

## Supplementary Materials

The following supporting information can be downloaded at https://www.mdpi.com/article/10.3390/jcm15041565/s1.

## Abbreviations

AUC, area under the concentration–time curve; DBS, dried blood spot; EDTA-2Na, ethylenediaminetetraacetic acid disodium salt; eGFR, estimated glomerular filtration rate; EVR, everolimus; Hct, hematocrit; IS, immunosuppressive drug(s); LC-MS/MS, liquid chromatography–tandem mass spectrometry; LSS, limited sampling strategy; MAPE, mean absolute percentage error; MF-CMS, microfluidic capillary microsampling system; MMF, mycophenolate mofetil; MPAG, mycophenolic acid β-D-glucuronide; MPA, mycophenolic acid; MPE, mean prediction error; MSW2™, Microsampling Wing MSW2™; mPSL, methylprednisolone; PK, pharmacokinetics; RMSE, root mean squared error; SOT, solid-organ transplantation; TAC, tacrolimus; TDM, therapeutic drug monitoring; TOX, toxicity study; VAMS, volumetric absorptive microsampling.
