# Supplementary material for: Clinical Application of Microvolume LC–MS/MS for Therapeutic Drug Monitoring of Immunosuppressants in Solid-Organ Transplant Recipients
Source: J Clin Med. 2026 Feb 16;15(4):1565. doi: 10.3390/jcm15041565 (PMC12941667; doi:10.3390/jcm15041565)
Supplement: Supplementary file 1 [file jcm-15-01565-s001.zip › jcm-4086029-supplementary (author proofed)/20251122 MSW2 Supplementary Figure1.pdf]

Fig. S1

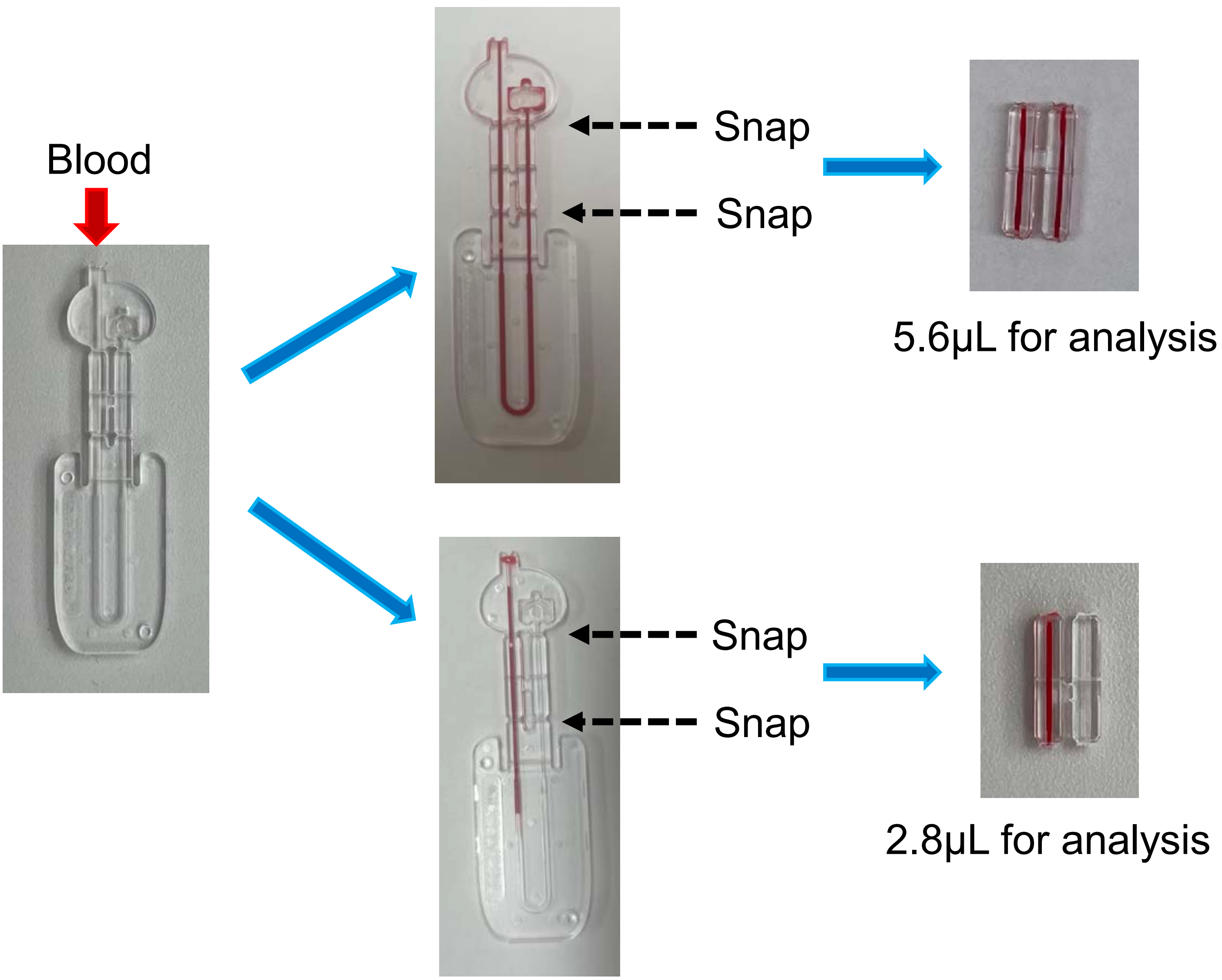

Fig S1. Methods of blood microsampling from fingertips using MSW2™

Patient fingertips are punctured with a lancet needle to create a small blood pool. Blood is drawn into the MSW2™ inlet port by capillary action. If 5.6 μL are to be collected (upper arrow), the segment is snapped in place, ensuring that blood is collected in the entire length of the U-shaped collection slot. If 2.8 μL are to be collected (lower arrow), blood is collected in only one side of the U-shaped blood collection slot and the segment is snapped at the designated point.
