# Supplementary material for: Clinical Application of Microvolume LC–MS/MS for Therapeutic Drug Monitoring of Immunosuppressants in Solid-Organ Transplant Recipients
Source: J Clin Med. 2026 Feb 16;15(4):1565. doi: 10.3390/jcm15041565 (PMC12941667; doi:10.3390/jcm15041565)
Supplement: Supplementary file 1 [file jcm-15-01565-s001.zip › jcm-4086029-supplementary (author proofed)/20251122 MSW2 Supplementary Figure2.pdf]

# Fig. S2

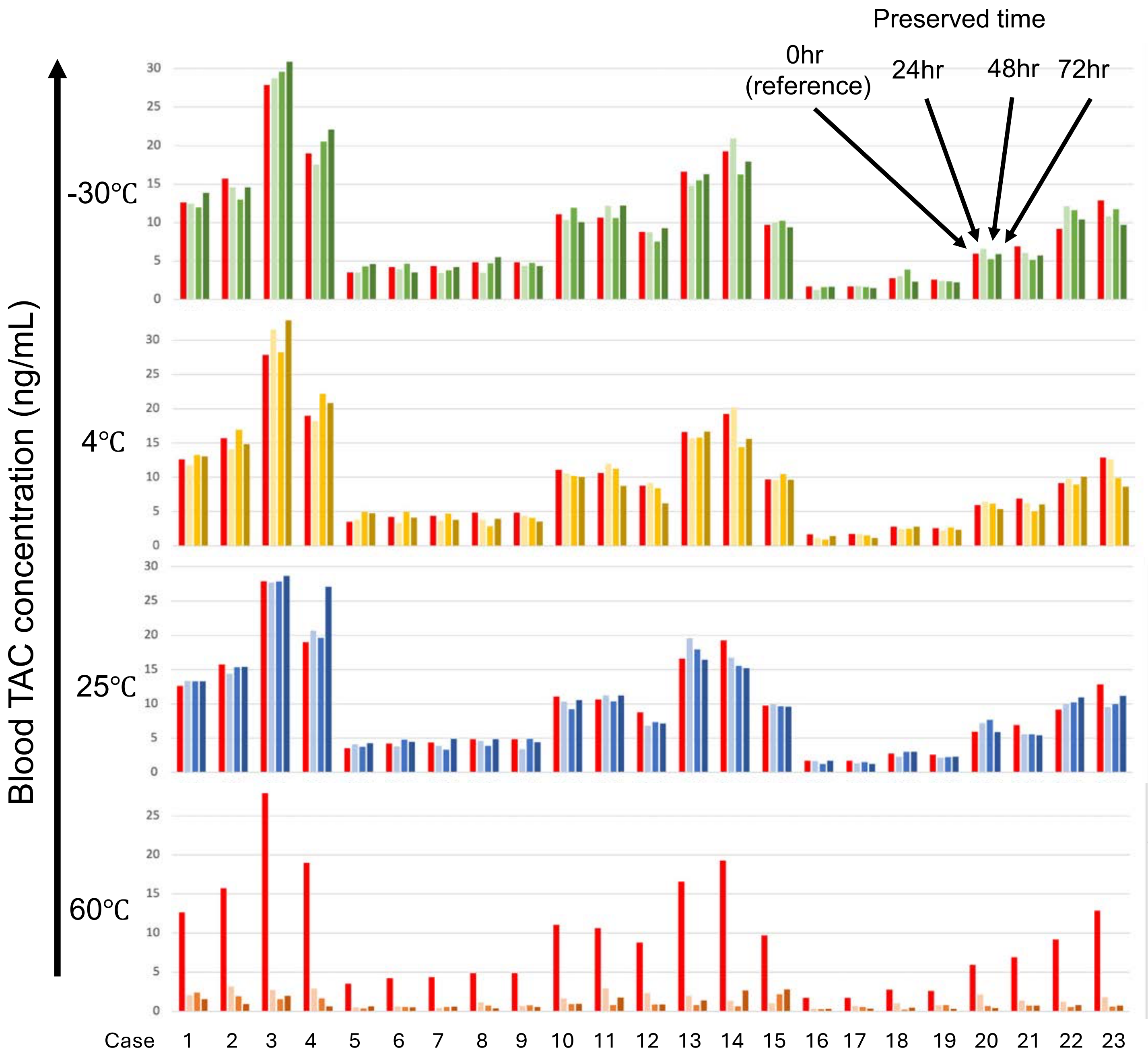


**Fig S2. Stability of microsampled blood under different temperatures and preservation periods for measurement of TAC concentration.**

Stability of microsampled blood in MSW2™ segments in Eppendorf tubes was examined after storage at various temperatures and for 24 h, 48 h, and 72 h. The change of TAC concentration ratio vs 0 h was evaluated up to 72 h at 4 storage temperatures (-30° C, 4° C, 25° C, and 60° C) in 23 participating cases. TAC: tacrolimus.
