# Supplementary material for: Clinical Application of Microvolume LC–MS/MS for Therapeutic Drug Monitoring of Immunosuppressants in Solid-Organ Transplant Recipients
Source: J Clin Med. 2026 Feb 16;15(4):1565. doi: 10.3390/jcm15041565 (PMC12941667; doi:10.3390/jcm15041565)
Supplement: Supplementary file 1 [file jcm-15-01565-s001.zip › jcm-4086029-supplementary (author proofed)/20251122 MSW2 Supplementary Figure4.pdf]

Fig. S4

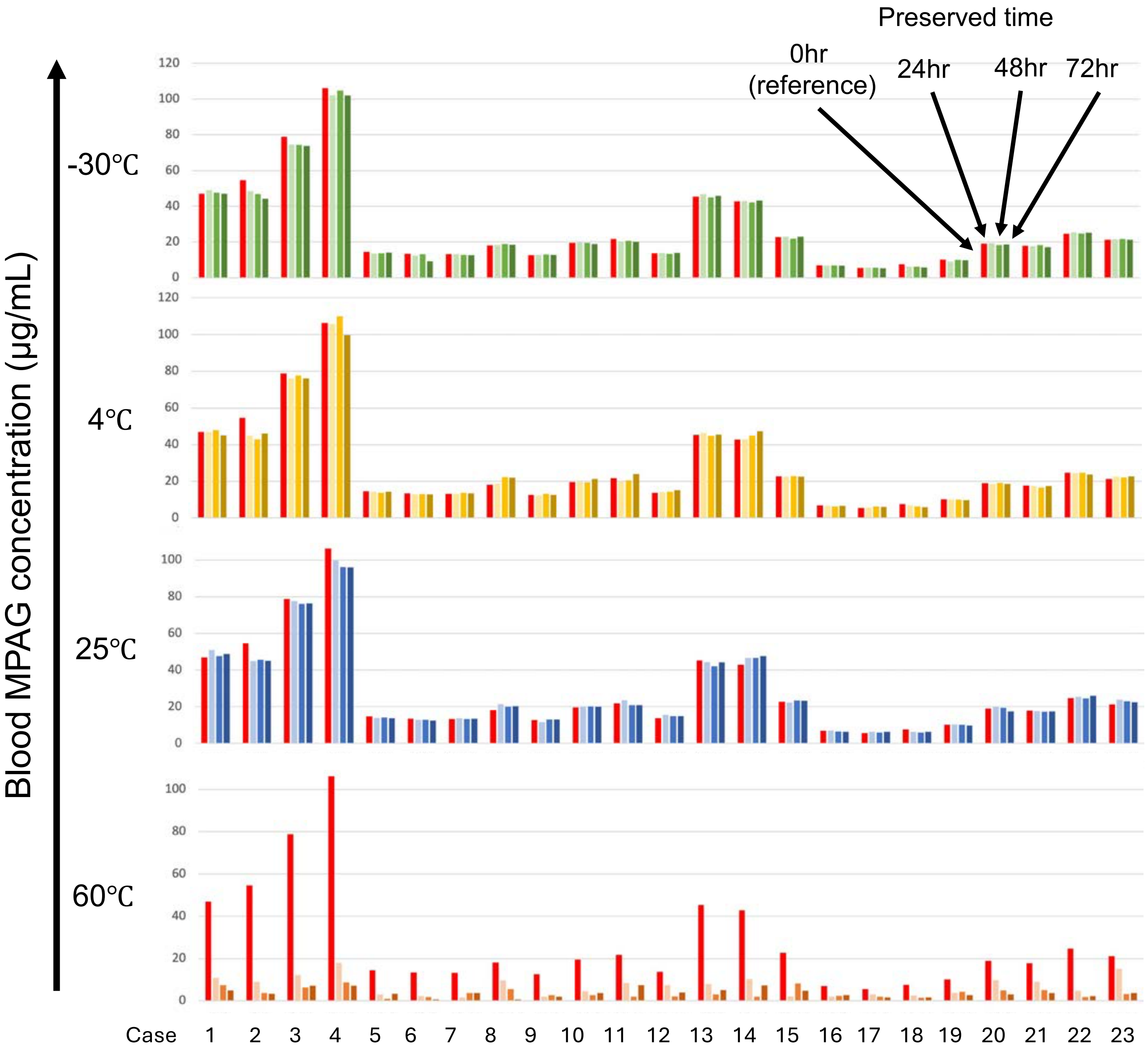

Fig S4. Stability of microsampled blood at different temperatures and preservation periods for measurement of MPAG concentrations.

Stability of microsampled blood in snapped MSW2™ segments in Eppendorf tubes was examined at various temperatures for 24 h, 48 h, and 72 h. The change of the MPAG concentration ratio relative to the 0-hour concentration was evaluated up to 72 h at 4 storage temperatures (-30° C, 4° C, 25° C, and 60° C) in 23 patients. MPAG: mycophenolic acid glucuronide.
