# Supplementary material for: Clinical Application of Microvolume LC–MS/MS for Therapeutic Drug Monitoring of Immunosuppressants in Solid-Organ Transplant Recipients
Source: J Clin Med. 2026 Feb 16;15(4):1565. doi: 10.3390/jcm15041565 (PMC12941667; doi:10.3390/jcm15041565)
Supplement: Supplementary file 1 [file jcm-15-01565-s001.zip › jcm-4086029-supplementary (author proofed)/〇20260129 MSW2 Supplementary Tables (author proofed).docx]

**Supplementary Table S1: Comparisons between plasma MPA and estimated plasma MPA**

| **Comparison** | **Passing-Bablok Regression** | | | **Bland-Altman Ratio %**  **Difference vs Average** | |
| --- | --- | --- | --- | --- | --- |
|  | **Slope (95% CI)** | **Intercept (95% CI)** | **τ value** | **Bias (95% LOA)** | **Bias SD** |
| estimated plasma MPA  vs plasma MPA | 0.971 (0.940 to 1.002) | -0.015 (-0.102 to 0.065) | 0.884 | 4.898 (-26.96 to 36.74) | 16.25 |
| estimated plasma MPA= blood MPA x 100 / (100-Hct) μg/mL | | | | | |

Abbreviations. Hct: hematocrit, LOA: limit of agreement, MPA: mycophenolic acid,

| **Collected volume for analysis** | 5.6μL | 2.8μL |
| --- | --- | --- |
| **No. of attempts** | 70 | 50 |
| **Success rate (Success / failure)** | 72.9 % (51 / 19) | 94.0 % (47 / 3) |
| **p value by chi-square test** | p=0.0032 | |

**Supplementary Table S2: Success rate of collecting blood from fingertips for measurement of drugs using MSW2™**
